# Supplementation with Matured Hop Bitter Acids Improves Cognitive Performance and Mood State in Healthy Older Adults with Subjective Cognitive Decline

**DOI:** 10.3233/JAD-200229

**Published:** 2020-06-30

**Authors:** Takafumi Fukuda, Tohru Ohnuma, Kuniaki Obara, Sumio Kondo, Heii Arai, Yasuhisa Ano

**Affiliations:** a KIRIN Central Research Institute, Kirin Holdings Company, Ltd., Kanagawa, Japan; b Department of Psychiatry, Juntendo University Faculty of Medicine, Tokyo, Japan; c Fukushima Healthcare Center, Osaka, Japan

**Keywords:** Attention, dietary supplements, hops, subjective cognitive decline

## Abstract

**Background:**

Prevention of age-related cognitive decline and depression is becoming urgent because of rapid growing aging populations. Effects of vagal nerve activation on brain function by food ingredients are inadequately investigated; matured hop bitter acid (MHBA) administration reportedly improves cognitive function and depression via vagal nerve activation in model mice.

**Objective:**

We investigated the effects of MHBA supplementation on cognitive function and mood state in healthy older adults with perceived subjective cognitive decline.

**Methods:**

Using a randomized double-blind placebo-controlled trial design, 100 subjects (aged 45–69 years) were randomly assigned into placebo (*n* = 50) and MHBA (*n* = 50) groups, and received placebo or MHBA capsules daily for 12 weeks.

**Results:**

Symbol Digit Modalities Test (SDMT) score assessing divided attention at week 12 was significantly higher (*p* = 0.045) and β-endorphin at week 12 was significantly lower (*p* = 0.043) in the subjects receiving MHBA. Transthyretin in serum, a putative mild cognitive impairment marker, was significantly higher at week 12 in the MHBA group than in the placebo group (*p* = 0.048). Subgroup analysis classified by the subjective cognitive decline questionnaire revealed that in addition to improved SDMT scores, memory retrieval assessed using the standard verbal paired-associate learning tests and the Ray Verbal Learning Test at week 12 had significantly improved in the subgroup with perceived subjective cognitive decline and without requirement for medical assistance in the MHBA group compared with that in the placebo group.

**Conclusion:**

This study suggested that MHBA intake improves cognitive function, attention, and mood state in older adults.

## INTRODUCTION

Dementia and cognitive decline are growing global concerns in today’s aging societies; however, there is a lack of promising drugs for their treatment. There is an increased focus on maintaining cognitive function in old age and delaying the onset of neurodegenerative diseases such as Alzheimer’s disease (AD) [1]. Recent studies have reported that early interventions in older people with subjective cognitive decline (SCD) in the absence of objective neuropsychological dysfunction are effective in preventing cognitive decline [2]. In the past two decades, methods for improving brain function via vagus nerve stimulation (VNS) have been developed. VNS is a novel treatment method for cognitive disorders [3, 4] and a Food and Drug Administration approved treatment for drug-resistant epilepsy and depression [5]. These VNS therapies require surgical implantation of a small device that provides electrical stimulation to the vagus nerve, thus, they have limited non-medical application. Recent studies suggest that a practical and non-invasive nutritional approach to vagal nerve stimulation can improve brain function.

Epidemiological studies indicate that moderate consumption of alcoholic beverages is beneficial to cognitive function [6, 7]. Resveratrol, a polyphenol present in red wine, has been reported to improve cognitive function in animal models as well as humans [8].

Previous studies demonstrate that iso-*α*-acids (IAAs), which are hop-derived bitter compounds commonly used to make beer, activate the vagal nerve system, resulting in the improvement of cognitive function and depression in the model mice [9, 10]. Additionally, mature hop bitter acids (MHBA), which have a chemical structure similar to that of iso-*α*-acids, act within the intestines to stimulate the vagus nerve and increase norepinephrine secretion in the brain, resulting in the improvement of cognitive function and depression in the model animals [11–14]. These reports suggest that activation of the vagal nerve by food ingredients can improve cognitive function and psychological state. A preliminary experiment by our research group demonstrated that MHBA supplementation (35 mg/day) improved subjects’ scores on the verbal fluency test and the Stroop test (color-word interference), which evaluated memory and attention in healthy middle-aged adults (45–64 years old) [15].

We conducted the present study in order to evaluate the effects of MHBA on human cognitive function and mood state in older adults with SCD. We used neuropsychological tests, questionnaires, and objective mental stress markers to assess cognitive function and mood states. We also measured putative mild cognitive impairment (MCI) markers in serum [16]. Furthermore, to evaluate the association of the level of SCD and the effectiveness of the MHBA supplement, the subjects were characterized by an SCD questionnaire (SCD-Q) [17, 18] and subgroup exploratory analyses were performed.

## MATERIALS AND METHODS

### Participants

Our study subjects were native Japanese participants. The following criteria were assessed during the screening session by questionnaire and clinical examination. The inclusion criteria were: 1) aged 45–69 years; 2) primary language Japanese; 3) participants with perceived SCD judged by the SCD-Q [17], that is, those who answered “YES” to at least one of the 24 questions. The exclusion criteria were: 1) visual or hearing impairment; 2) suspected dementia based on Mini-Mental State Examination (MMSE) score <24; 3) history of cranial nerve disease; 4) current hormonal treatment; 5) irregular lifestyle; 6) habitual consumption of beer-based drinks (including non-alcoholic drinks); 7) high habitual consumption of alcohol (>20 g/day); 8) smoking habit; 9) participation in the same neuropsychological tests within the past year; 10) regular consumption of drugs affecting cognitive function; 11) history of clinical sleep-related disorders; 12) blood donation within the past three months; 13) participation in any other clinical trials; 14) pregnant or breastfeeding; 15) allergy to the test food; 16) diagnosed dry mouth; 17) diagnosed arrhythmia.

### MHBA and placebo supplements

MHBA were prepared as described previously [14, 19]. The same MHBA test samples were used in a previous clinical study [15]. MHBA were composed of 4’-hydroxyallohumulinones [13], 4’-hydroxyalloisohumulones [11], tricyclooxyisohumulones A [20], hulupones, and humulinones [13], which are oxidants of *α* and β-acids. Test capsules containing 35 mg of MHBA or 35 mg of dextrin (placebo) were prepared by Kirin Holdings Co. Ltd (Tokyo, Japan). The nutritional composition of the test capsules are presented in Table 1. We confirmed that there were no discernible differences between the appearance and taste of the MHBA and placebo capsules. Test subjects ingested the capsules orally with water every day for twelve weeks. Compliance was monitored by counting the number of capsules remaining at the end of the experiment.

**Table 1:** Nutritional composition of matured hop bitter acids (MHBA) and placebo capsules (amount per dose/day)

	Placebo capsule	MHBA capsule
MHBA (mg)	0	35
Moisture (mg)	73	61
Protein (g)	0.2	0.3
Carbohydrate (g)	0.6	0.5
Energy (kcal)	3.4	3.5
Sodium (mg)	0.07	0.12

### Trial design

**Fig. 1:**
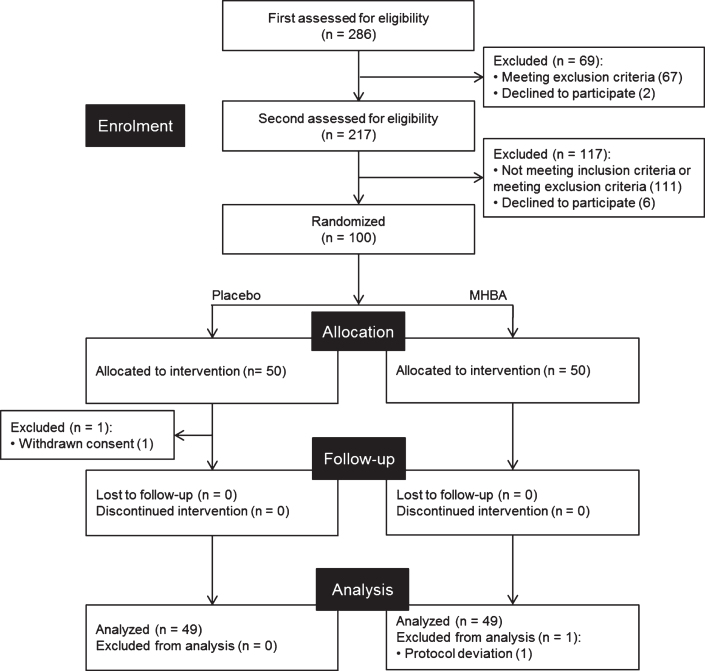
CONSORT diagram. Out of the 286 subjects who were screened, 100 subjects were included in this study. These subjects were randomly allocated to placebo (*n* = 50) and MHBA (*n* = 50) groups. One subject in the placebo group withdraw consent and one subject in the MHBA group was excluded from analysis, leaving 49 subjects for analysis in each group.

This was a randomized double-blind placebo-controlled parallel-group comparative study carried out over twelve weeks in Osaka, Japan. Figure 1 shows the screening procedures we employed to find 100 participants. Since this exploratory study was the first clinical trial to evaluate the potential of MHBA for cognitive function, an appropriate sample size was calculated based on previous human trials evaluating the effects of food ingredients on cognitive function [21–23]. Primary screening included questionnaires for inclusion/exclusion criteria, MMSE, and clinical examination for safety assessments. Secondary screening included neuropsychological tests, autonomic nerve measurement, and subjective psychological assessments. Individuals who fulfilled the eligibility criteria were recruited and randomly allocated to either the MHBA or placebo group in a 1:1 ratio by a third-party allocator using a computer program. The allocator was not involved in determining eligibility, data collection, or analysis. Research staff, outcome assessors and participants were blinded to the group allocation until the data analysis was complete.

Outcome assessments were performed at the baseline (week zero) and week twelve of the experiment. Participants were instructed to maintain their regular lifestyle, including eating habits, and to avoid using drugs or supplements that could affect cognitive function.

Neuropsychological tests were conducted under rigorously controlled conditions. In brief, on the day of the neuropsychological test, participants were instructed to abstain from consuming food and beverages other than water for 4 h before the tests. Data were collected at the DRC Co., Ltd. (Osaka, Japan), and the study was performed by the contract research organization TTC Co., Ltd. (Tokyo, Japan).

### Neuropsychological tests

Neuropsychological tests to evaluate attention and memory were conducted by trained assessors who were blinded to the group allocation.

Attention was evaluated using the Clinical Assessment for Attention (CAT) [24]. The CAT consists of four tests. The Symbol Digit Modalities Test (SDMT) is used to assess divided attention. The SDMT is a cognitive test that consists of a sheet of paper with an answer key at the top (nine symbols and their correspondent numbers from 1 to 9) and a series of 120 random repetitions of those symbols [24]. The subject is required to enter the numbers that correspond to each symbol within a time limit of 90 s. The cancellation task is used to assess selective attention and requires subjects to delete targets (sign 1, sign 2, number “3”, and Japanese “ka”) as fast as possible in interference stimulus. The memory updating test, in which participants were required to repeat the last three or four spans to the assessor was performed to assess working memory. The Position response test, in which Japanese words for “upper”, “middle”, or “lower” were printed in random positions, required participants to identify the position of the word instead of the meaning of the word.

Memory retrieval was evaluated using the Rey Auditory Verbal Learning Test (RAVLT) and S-PA. The word recall test was conducted according to the RAVLT [25]. Participants were presented with 15 words (List A) and asked to verbally recall them immediately and again after 5 min (total immediate memory (TIM); scores range from 0 to 75). Next, 15 different words (List B) were presented and participants were asked to verbally recall them as an interference task. Following the interference task, participants were asked to verbally recall List A immediately and then again after 20 min (T7). The number of forgotten words (T7–T6) was also evaluated. The verbal and visual paired-associate learning test was conducted according to the S-PA [26], in which subjects were presented pairs of words that were semantically unrelated. Subsequently, subjects were presented with one of each pair of words and were required to correctly recall the other. The trials were conducted three times and the total score was evaluated in the S-PA range of 0–30. A figural memory test was conducted according to the Wechsler Memory Scale-Revised (WMS-R) [27], in which subjects were required to memorize meaningless shapes. Subsequently, subjects were presented some meaningless shapes and were required to choose the memorized one.

### Mental stress markers in saliva

To measure stress levels, saliva was collected at the baseline (week zero) and week twelve of the experiment, before and after the neuropsychological tests using salivette tubes (Sarstedt, Tokyo, Japan). Salivary β-endorphin, cortisol, chromogranin A (CgA) and *α*-amylase were assayed using commercially available kits (β-Endorphine S-1134 kit, Peninsula Laboratories Inc., Belmont, CA, USA; Cortisol, EIA Kit, Salimetrics Inc., State College, PA, USA; Chromogranin A EIA kit, Yanaihara Institute, Shizuoka, Japan; *α*-Amylase Assay Kit, Salimetrics Inc., State College, PA, USA).

### Mental stress, mood state, and subjective cognitive function assessments

Anxiety traits that reflect long-term anxiety were evaluated prior to neuropsychological testing using the State-Trait Anxiety Inventory (STAI)-FormX-2 (scores range from 20 to 80) [28]. Subjective cognitive function was evaluated using the Metamemory in Adulthood Questionnaire (MIQ-A) which consists of six sub-categories; Change, Task, Capacity, Anxiety, Strategy, and Locus [29]. We also evaluated the change of subjective mood state during the neuropsychological tests using STAI-FormX-1 because anxiety-state may affect the neuropsychological test performance [30–32]. Subjective drowsiness was evaluated before and after the neuropsychological tests using the Karolinska Sleepiness Scale-Japanese version (KSS-J) [33].

### Indicators in serum

Blood samples were collected after neuropsychological testing. Transthyretin (TTR), Apolipoprotein AI (ApoA1), and Complement 3 (C3) in serum were assayed using commercially available kits (N-assay, TIA preALB, TIA ApoA1, and TIA C3-SH, Nittobo Medical Inc., Fukushima, Japan) as markers of MCI. [16]. Brain-derived neurotrophic factor (BDNF) was quantified using Human/Mouse BDNF DuoSet ELISA (R & D Systems, Inc., Minneapolis, MN, USA). Cholecystokinin (CCK) was quantified using Human/Mouse/Rat CCK Enzyme Immunoassay Kit (RayBiotech, Peachtree Corners, GA, USA). These two indicators were evaluated to elucidate the underlying mechanism exploratory.

### A posteriori subgroup analysis

Early diagnosis and treatments for the prevention of dementia are gathering increased attention. In particular, adults with perceived SCD are regarded as important candidates for early intervention. Previous research indicates that SCD can be divided into two groups: Those with perceived subjective cognitive decline (SDC-P) who have not sought medical assistance for their complaints, and those with clinical subjective cognitive decline (SCD-C) who have sought medical assistance [34]. Our exploratory study focused on these two SCD groups and subgroup analysis was performed to evaluate the effects of MHBA supplementation on cognitive function. Subjects were classified by their answers to the SCD-Q questions: 1) “Do you perceive memory or cognitive difficulties?”, 2) “Would you ask a doctor about these difficulties?”, and 3) “In the last two years, has your cognition, or memory declined?” [17, 18]. Subjects who answered “YES” to all three questions were categorized as SCD-C and subjects who answered “YES” to the first and third questions and “NO” to the second question were categorized as SCD-P.

### Statistical analysis

Data analysis was performed by a member of the research staff blinded according to a predefined plan. The results were expressed as a mean with standard deviation. Statistical comparisons were performed using IBM SPSS Statistics 23 (IBM, New York, NY, USA) and BellCurve for Excel (Social Survey Research Information Co., Ltd., Tokyo, Japan). Comparisons of all results, except MIA-Q, KSS-J, and STAI, were conducted using paired (baseline and week twelve scores) or unpaired (between-group differences in mean week twelve scores and changes in score from the baseline) *t* tests. The MIA-Q, KSS-J, and STAI results were analyzed using Wilcoxon signed-rank test (baseline and week twelve scores) or Mann–Whitney *U* tests (between-group differences in mean week twelve scores and changes in score from the baseline). All *p*-values < 0.05 were considered statistically significant. Furthermore, preliminary multiple comparisons were performed on neuropsychological tests using z scores. Statistical analysis predefined in the protocol was conducted by the contract research organization TTC Co., Ltd. (Tokyo, Japan) and the funder was not involved in the statistical analysis.

### Standard protocol approvals, registrations, and patient consents

The study was conducted in accordance with the Declaration of Helsinki and Ethical Guidelines for Medical and Health Research Involving Human Subjects and was approved by the ethics committee of Kensho-kai (Osaka, Japan). Written informed consent was obtained from all participants. The study was registered on January 21, 2019 in the database of the University Hospital Medical Information Network (UMIN) prior to subject enrollment (Registration No. UMIN000035601).

## RESULTS

### Subject characteristics

Participants were screened between January and April 2019 and the experiment was conducted from April to July 2019. Out of the 286 candidates from the volunteer bank, a total of 100 participants were recruited; one participant withdrew consent during the trial. Thus, 99 participants completed the twelve-week study (Fig. 1). One participant was excluded from the analysis according to the exclusion criterion: regular consumption of drugs affecting cognitive function during the trial. Therefore, a total of 98 participants’ results were analyzed and their characteristics are presented in Table 2. There were no significant differences in ingestion compliance between placebo or MHBA groups (>95.3% compliance). No serious adverse effects were observed in either group.

**Table 2:** Characteristics of the study participants at baseline

Characteristics	Placebo	MHBA	*p*
	(*n* = 49)	(*n* = 49)
Age	53.3±4.9	54.6±6.3	0.25
Male/female	21/28	20/29	1.00
MMSE score (/30)	28.5±1.2	28.3±1.2	0.55
SCD-Q a) (YES/NO)	46/3	40/9	0.12
SCD-Q b (YES/NO)	18/31	16/33	0.83
SCD-Q c) (YES/NO)	48/1	44/5	0.21
SCD-Q score (/24)	15.5±4.5	14.7±5.7	0.41
Employed/unemployed	39/10	40/9	1.00

Data represent mean±SD. *p*-values were calculated by unpaired *t* test for age, MMSE score, and SCD-Q score. *p*-values for male/female, SCD-Q a)–c), and employed/unemployed were calculated using *χ*^2^ test. MHBA, matured hop bitter acids; MMSE, Mini-Mental State Evaluation; SCD-Q, subjective cognitive decline questionnaire.

### Primary outcomes

#### Neuropsychological tests (attention)

**Fig. 2:**
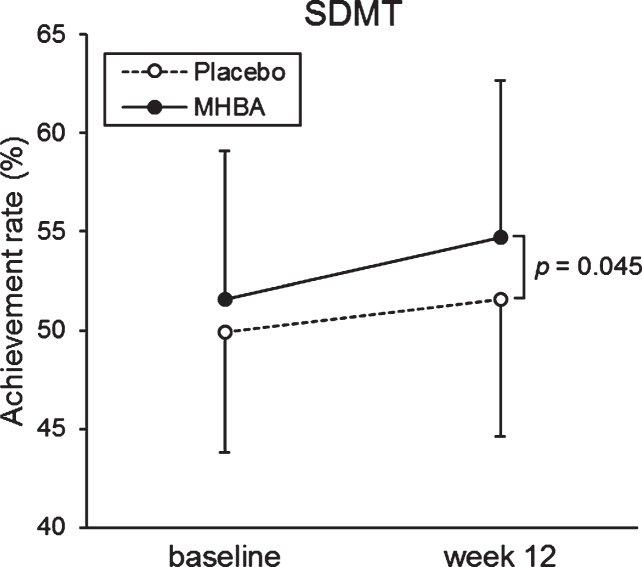
The mean values of SDMT at baseline and week twelve. The solid line indicates the MHBA group (*n* = 49) and the dotted line indicates the placebo group (*n* = 49). Data points represent means and error bars indicate SD. *p*-value shows between-group differences performed using unpaired *t* tests.

The primary endpoints of attention at the baseline and week twelve are summarized in Table 3. The week twelve of SDMT scores were significantly higher in the MHBA group compared to the placebo group (*p* = 0.045) (Fig. 2). When comparing the baseline (week zero) and week twelve CAT neuropsychological tests, we observed a significant decrease in time to accomplish the task for both the Visual Cancellation test and the Position response test. We also observed higher SDMT scores in both groups, with a greater increase in the MHBA group. There were no significant differences in z score that integrated attention scores (z score difference = 0.08; *p* = 0.48 at week twelve and = 0.15; *p* = 0.18 in the change from baseline to week twelve).

**Table 3:** Mean scores for attention performance

	Group	Baseline		Week 12	
		mean±SD	*p*	mean±SD	*p*
SDMT (achievement rate [% ])					
	Placebo	49.9±6.1	0.23	51.6±7.0^*^	0.045
	MHBA	51.6±7.5		54.7±8.0^**^#
Visual cancellation (time to accomplish the task [s])					
Sign 1	Placebo	55.9±12.5	0.51	48.8±8.4^**^	0.67
	MHBA	57.7±14.4		48.1±6.9^**^
Sign 2	Placebo	60.6±12.4	0.83	53.3±10.1^**^	0.53
	MHBA	61.1±10.4		52.1±8.2^**^
Number “3”	Placebo	94.6±15.7	0.91	86.0±12.8^**^	0.43
	MHBA	94.2±15.7		84.0±12.9^**^
Japanese “ka”	Placebo	113.0±16.6	0.97	104.0±15.0^**^	0.65
	MHBA	113.1±17.4		102.6±16.6^**^
Position response test (time to accomplish the task [s])					
1st	Placebo	75.2±14.6	0.56	68.5±10.8^**^	0.70
	MHBA	76.9±14.1		67.7±9.8^**^
2nd	Placebo	71.0±19.7	0.99	65.3±11.0^**^	0.91
	MHBA	71.1±14.1		65.1±10.1^**^
Memory updating (achievement rate [% ])					
3 span	Placebo	83.7±15.7	0.39	85.8±14.1	0.59
	MHBA	81.1±13.2		84.3±13.9
4 span	Placebo	68.4±21.9	0.54	70.9±21.0	0.59
	MHBA	65.7±21.0		68.8±19.0

Data represent mean±SD. *p*-values from unpaired *t* test; (Placebo versus MHBA) are presented. MHBA group (*n* = 49); Placebo group (*n* = 49); ^*^p < 0.05; ^**^p < 0.01 (paired *t* test compared with baseline); ^#^*p* < 0.05 (unpaired *t* test; Placebo versus MHBA). MHBA, matured hop bitter acids; SDMT, Symbol Digit Modalities Test.

#### Neuropsychological tests (memory)

The primary memory endpoints at the baseline and week twelve are presented in Table 4. T6–T5 scores are not reported for two subjects (subject 1 in both groups) because they did not follow the rules of the test. Week twelve S-PA and TIM scores significantly increased in both groups when compared with their baseline scores. The week twelve T6–T5 scores for the RAVLT were significantly higher than the baseline scores in the placebo group. The TIM scores at week twelve were higher in the placebo group than in the MHBA group (*p* = 0.084). There were no significant differences in z score that integrated memory scores (z score difference=–0.17; *p* = 0.25 at week twelve and = –0.09; *p* = 0.48 in the change from baseline to week twelve).

**Table 4:** Mean scores for memory performance

	Group	Baseline		Week 12	
		mean±SD	*p*	mean±SD	*p*
S-PA					
Unrelated pairs	Placebo	12.0±6.6	0.33	16.5±7.2^**^	0.43
	MHBA	10.7±6.5		15.2±8.4^**^
RAVLT					
TIM	Placebo	42.0±7.9	0.30	46.1±8.6^**^	0.084
	MHBA	40.0±11.0		42.6±11.0^**^
T6–T5	Placebo	–2.3±2.4	0.55	–1.4±1.9^*^	0.24
	MHBA	–2.5±2.0		–1.9±2.3
T7–T6	Placebo	–0.2±2.1	0.63	–0.6±1.9	0.28
	MHBA	–0.4±2.1		–0.2±2.2
WMS-R					
Figural memory	Placebo	7.0±1.6	0.90	7.4±1.2	0.94
	MHBA	7.1±1.5		7.4±1.5

Data represent mean±SD. *p*-value from unpaired *t* test; (Placebo versus MHBA) are presented. MHBA group (*n* = 49); Placebo group (*n* = 49); ^*^p < 0.05, ^**^p < 0.01 (paired *t* test compared with baseline). T6–T5 score were missing for two subjects (MHBA: 1 and Placebo: 1, respectively). MHBA, matured hop bitter acids; S-PA, Standard verbal paired-associate learning test; WMS-R, Wechsler Memory Scale-Revised; RAVLT, Rey Auditory Verbal Learning Test; TIM, Total Immediate Memory.

#### Stress conditions

**Fig. 3:**
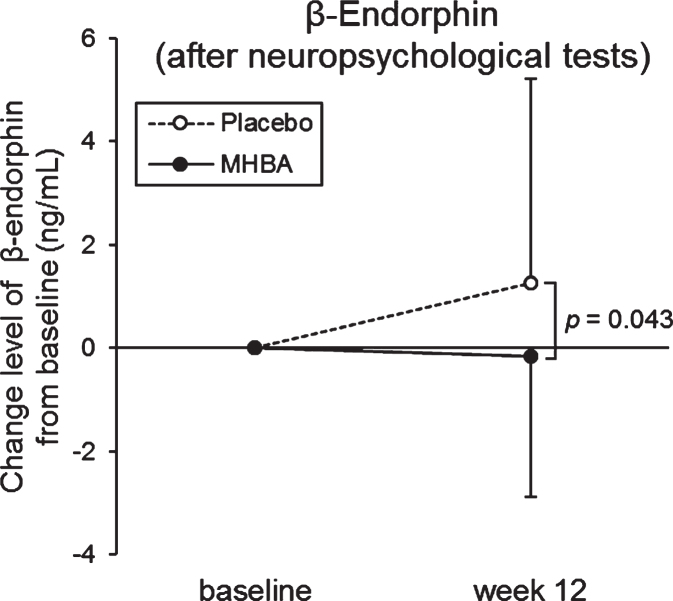
Changes in β-Endorphin levels at baseline and week twelve (after the neuropsychological tests). The solid line indicates the MHBA group (*n* = 49) and the dotted line indicates the placebo group (*n* = 48). Data points represent means and error bars indicate SD. *p*-value shows between-group differences performed using unpaired *t* tests.

To evaluate the level of stress, cortisol, CgA, *α*-amylase, and β-endorphin levels in saliva were measured at the baseline and week twelve and analyzed (Supplementary Table 1). Enough saliva for ELISA could not be collected from three subjects in the placebo group, therefore the following data were missing: three subjects for β-endorphin (before neuropsychological tests), one subject for β-endorphin (after neuropsychological tests), two subjects for cortisol (before neuropsychological tests), one subject for CgA, and one subject for *α*-amylase (before neuropsychological tests). Changes in β-endorphine levels from baseline to week twelve after neuropsychological tests were significantly lower in the MHBA group than in the placebo group (*p* = 0.043) (Fig. 3). Furthermore, in the MHBA group, cortisol levels after neuropsychological tests and amylase levels before neuropsychological tests were significantly decreased at week twelve compared with levels at the baseline. There were no significant changes in cortisol and amylase levels observed in the placebo group.

#### Subjective psychological assessment

**Fig. 4:**
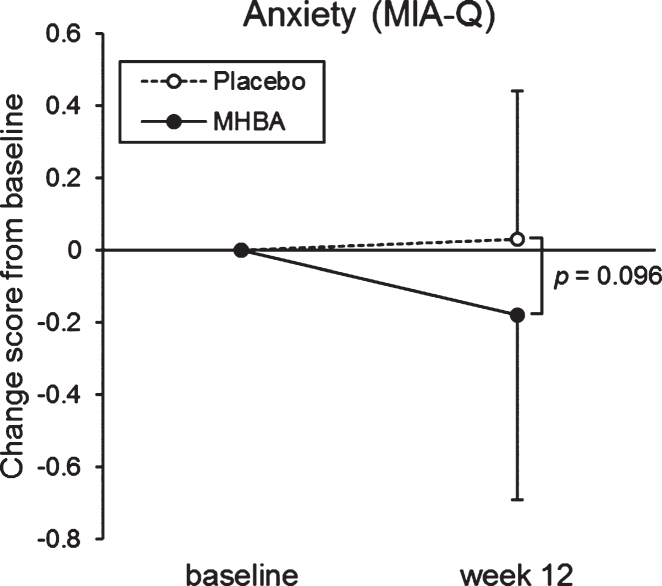
Changes in anxiety score in the MIA-Q at baseline and week twelve. The solid line indicates the MHBA group (*n* = 49) and the dotted line indicates the placebo group (*n* = 49). Data points represent means and error bars indicate SD. *p*-value shows between-group differences performed using Mann–Whitney *U* tests.

The change in score from baseline to week twelve in the “Anxiety” sub-category of the MIA-Q was tend to be lower in the MHBA group than the placebo group (*p* = 0.096, Supplementary Table 2, Fig. 4). In the “Task” sub-category, week twelve scores were significantly higher than the baseline scores in the MHBA group (*p* = 0.005), but not in the placebo group.

Week twelve anxiety-state STAI scores (after neuropsychological tests) significantly decreased in both groups compared to their baseline scores, but there was no difference in scores between the MHBA and placebo groups (Supplementary Table 3).

#### Indicators in serum

The results of indicators in serum are presented in Supplementary Table 4. The level of TTR at week twelve was significantly higher in the MHBA group than the placebo group (*p* = 0.048).

#### Subgroup analysis classified by the SCD-Q

**Fig. 5:**
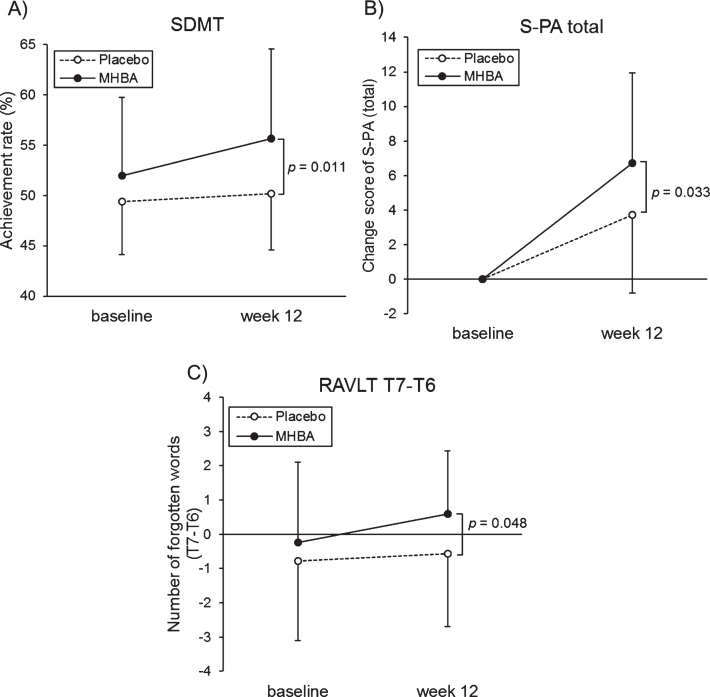
A) The mean values of SDMT at baseline and week twelve, B) changes in unrelated words scores of the S-PA from baseline, C) the mean values of T7–T6 in the RAVLT at baseline and week twelve in the SCD-P. The solid line indicates the MHBA group (*n* = 22) and the dotted line indicates the placebo group (*n* = 28). Data points represent means and error bars indicate SD. *p*-values show between-group differences performed using unpaired *t* tests.

The characteristics of the SCD-C and SCD-P subgroups are presented in Supplementary Table 5. In the SCD-P subgroup, the SDMT score at week twelve was significantly higher in the MHBA group compared to the placebo group (*p* = 0.011) (Fig. 5A). Furthermore, changes in the S-PA scores from the baseline to week twelve were significantly greater in the MHBA group compared to the placebo group (*p* = 0.033) (Fig. 5B). The MHBA group also remembered significantly fewer words (T7–T6) than the placebo group at week twelve compared to the baseline (*p* = 0.048) (Fig. 5C). Z score that integrated attention and memory scores tended to be higher in MHBA group than placebo group (z score difference = 0.21; *p* = 0.11 at week twelve and = 0.26; *p* = 0.078 in the change from baseline to week twelve). In the SCD-C subgroup, there were no significant differences between the MHBA and placebo groups in the neuropsychological tests.

## DISCUSSION

In this randomized double-blind placebo-controlled trial, we evaluated the effects of twelve weeks of MHBA supplementation (35 mg/day) in healthy older adults. We observed improved attention (assessed by the SDMT) and reduced stress after neuropsychological tests (assessed by β-endorphin) in the participants who received MHBA supplements.

SDMT performance is an extremely sensitive predictor of progression from amnestic MCI to dementia [35]. The SDMT assesses mental processing speed and concentration functions, in addition to divided attention [36]. Previous imaging studies reported that cognitive operations in multiple brain areas including the bilateral middle frontal, inferior frontal and lingual gyri, superior parietal lobule, declive, cuneus, precuneus, and dorsolateral prefrontal cortex (dlPFC) are highly involved in the performance of the SDMT [37, 38]. Our results are consistent with reports from a previous clinical trial that MHBA improved verbal fluency and Stroop test (color-word interference) performance, both of which reflect dlPFC function [15].

Previous studies also report that VNS therapy improved SDMT performance in refractory depression patients [39]. Additionally, locus-noradrenergic systems are responsible for cognitive processes including attention [40], and methylphenidate, a stimulant drug with dopamine and norepinephrine reuptake inhibition properties, improves attention in healthy volunteers [41]. Thus, the underlying mechanism for improved attention in our MHBA group may be increased norepinephrine levels via MHBA-induced VNS, as suggested by preclinical studies.

MHBA supplementation reduced the levels of β-endorphin after the neuropsychological tests. β-Endorphin is a marker of hypothalamic-pituitary-adrenal (HPA)-axis activity [42], which is used to evaluate psychological stress (e.g., stress caused by public speaking, watching stressful videos, or taking examinations) [43–45]. Additionally, patients suffering from depression are reported to have higher β-endorphin levels [46, 47]. Cortisol, another marker for stress reactions caused by the HPA-axis [48], and β-endorphin levels were significantly lower after neuropsychological tests in subjects who received the MHBA supplement (Supplementary Table 1). *α*-Amylase and CgA reflect sympathetic-adrenal-medullary (SAM)-axis response to acute stress [49, 50]. *α*-amylase and CgA levels were not affected by the MHBA supplementation. This could be because the stress markers regulated by the SAM-axis decrease back to the baseline faster than those regulated by the HPA-axis [51]. Saliva was collected 15 minutes before and after the neuropsychological test, therefore our study design did not detect any effect of MHBA on stress response using SAM-axis markers. In future studies, to evaluate the effect of MHBA on mental stress in more detail, it would be necessary to set more frequent saliva collection time points.

The Anxiety sub-category of the MIA-Q [29], which evaluated both the introspective knowledge of one’s own memory capabilities (and strategies that can aid memory) and the processes involved in memory self-monitoring, tended to improve in the MHBA group compared with the placebo group. This result was consistent with our previous study in which daily anxiety-state (evaluated by questionnaire) was improved by MHBA supplementation [15]. Anxiety-trait was a robust indicator of the subject’s personality and changed little before and after the intervention. Anxiety-state, which reflects transient anxiety to the neuropsychological tests, and autonomic nerve activity were assessed before and after the neuropsychological tests because anxiety-state may affect the neuropsychological test performance [30–32]. We confirmed that there were no significant differences between the groups.

In the present study, putative MCI markers were evaluated and the level of TTR, which acts as an Aβ protein scavenger, was significantly higher at week twelve in the MHBA group than in the placebo group (*p* = 0.048) (Supplementary Table 4) [16]. A previous study by our research group reported supplementation of IAAs, the bitter component derived from hops, induced TTR expression in AD model mice [52]. Thus, the elevated TTR levels observed in the present study indicate that MHBA supplementation may be a viable AD therapy, but longer trials must be considered.

Furthermore, previous study indicated that MHBA improved the energy metabolism in over weighted subjects [53]. Since obesity is known risk factor for dementia [54], MHBA may prevent dementia by improving metabolic function. In the future study, we will design the clinical trial to evaluate the effect of MHBA on the association of energy metabolism with cognition.

Previous studies reported that SCD groups were heterogeneous (i.e., memory complaints were associated with physical health problems, depression, feelings of underachievement, low perceived self-efficacy, and high neuroticism). In particular, it has been reported that the characteristics of subjects differ depending on whether studies on SCD were recruited from community volunteers or clinical patients [55]. Community-based SCD subjects who perceived self-cognitive decline but did not seek medical help were classified as SCD-P and clinic-based SCD subjects who sought medical help were classified as SCD-C. In the current study, SCD-C and SCD-P were classified by three questions in the SCD questionnaire [17, 18]. Previous reports indicate that SCD-P classification suggests that AD-like pathology is less advanced than SCD-C. For example, hippocampal atrophy was specifically associated with medical help-seeking participants (SCD-C) [55]. Our subgroup analysis revealed that SDMT and memory retrieval scores were improved by the intake of MHBA in the SCD-P group. Thus, we hypothesize that early intervention with MHBA supplementation (before the onset of hippocampal atrophy) could be more beneficial for the improvement of brain function, but further study based on functional MRI is required.

We acknowledge the limitations of this study. First, as the result of performing the analysis considering multiplicity by the integrated analysis using z scores, there were no significant differences between the groups. In the future, it is necessary to verify the effect of MHBA on cognitive function from multiple study results by meta-analysis. Furthermore, we could not evaluate whether MHBA supplementation prevented age-related cognitive decline due to the relatively short study length. Future long-term studies would be required to assess the effects of MHBA supplementation on aging-related changes in order to avoid the learning and ceiling effects caused by repetition of the same test. Additionally, the mechanism underlying the cognition-improving effects of MHBA via vagus nerve has not been confirmed, therefore further study is required to elucidate the mechanism.

In conclusion, the present study results showed that MHBA supplementation improved mental processing speed, attention, and concentration and reduced mental stress after intellectual work in healthy adults aged 45–69 years with SCD. In particular, early intervention through MHBA supplementation in persons with SCD could be successful in improving cognitive function.

## Supplementary Material

Supplementary Material
