# Supplementary material for: Supplementation with Matured Hop Bitter Acids Improves Cognitive Performance and Mood State in Healthy Older Adults with Subjective Cognitive Decline
Source: J Alzheimers Dis. 2020 Jun 30;76(1):387–98. doi: 10.3233/JAD-200229 (PMC7369117; doi:10.3233/JAD-200229)
Supplement: Supplementary Material [file jad-76-jad200229-s001.pdf]

Supplementary Material

Supplementation with Matured Hop Bitter Acids Improves Cognitive Performance and Mood State in Healthy Older Adults with Subjective Cognitive Decline

Supplementary Table 1. Stress markers in saliva

		group	baseline		week 12	
			mean ± SD	*p*	mean ± SD	*p*
Cortisol (μg/mL)	before tests	Placebo	19.27 ± 9.58	0.14	19.15 ± 10.13	0.40
		MHBA	16.74 ± 6.82		17.66 ± 6.61	
	after tests	Placebo	17.90 ± 7.62	0.80	16.57 ± 8.05	0.26
		MHBA	18.55 ± 15.92		14.78 ± 7.61*	
Chromogranin A (pmol/mL)	before tests	Placebo	14.74 ± 16.18	0.86	19.34 ± 24.45	0.18
		MHBA	14.23 ± 12.08		13.94 ± 13.84	
	after tests	Placebo	13.08 ± 15.69	0.42	19.35 ± 18.96**	0.55
		MHBA	10.73 ± 13.03		16.77 ± 23.62*	
α-Amylase (U/μL)	before tests	Placebo	0.26 ± 0.36	0.63	0.21 ± 0.20	0.58
		MHBA	0.23 ± 0.17		0.20 ± 0.13*	
	after tests	Placebo	0.29 ± 0.30	0.64	0.26 ± 0.27	0.49
		MHBA	0.27 ± 0.20		0.23 ± 0.16	
β-endorphin (ng/mL)	before tests	Placebo	3.97 ± 6.80	0.68	3.70 ± 3.63	0.84
		MHBA	3.47 ± 4.82		3.51 ± 5.08	
	after tests	Placebo	3.11 ± 3.13	0.65	4.37 ± 4.20*	0.11
		MHBA	3.41 ± 3.38		3.26 ± 2.39	

Data represent mean ± SD. *p*-value from unpaired *t* test; (Placebo versus MHBA) are presented. MHBA group (n = 49); Placebo group (n = 49); β-endorphin data were missing for three subjects (Placebo: 3), cortisol data were missing for two subjects (Placebo: 2), chromogranin A data were missing for one subject (Placebo: 1) and α-amylase data were missing for one subject (Placebo: 1). $*p < 0.05$, $**p < 0.01$ (paired *t* test compared with baseline). MHBA, matured hop bitter acids.

Supplementary Table 2. Mean scores of Metamemory in Adulthood questionnaire (MIA-Q).

	group	baseline		week 12	
		mean ± SD	*p*	mean ± SD	*p*
**MIA-Q**					
Change	Placebo	1.80 ± 0.44	0.54	1.90 ± 0.50	0.68
	MHBA	1.85 ± 0.56		2.01 ± 0.69	
Task	Placebo	3.98 ± 0.54	0.52	4.09 ± 0.56	0.66
	MHBA	3.91 ± 0.45		4.06 ± 0.45**	
Capacity	Placebo	2.33 ± 0.41	0.48	2.52 ± 0.54*	0.72
	MHBA	2.37 ± 0.59		2.56 ± 0.56**	
Anxiety	Placebo	3.64 ± 0.66	0.89	3.67 ± 0.62	0.073
	MHBA	3.58 ± 0.75		3.40 ± 0.77	
Strategy	Placebo	2.83 ± 0.76	0.29	2.89 ± 0.76	0.54
	MHBA	2.62 ± 0.50		2.77 ± 0.69	
Locus	Placebo	3.01 ± 0.55	0.23	3.11 ± 0.54	0.51
	MHBA	3.15 ± 0.54		3.18 ± 0.49	

Data represent mean ± SD. *p*-value from Mann–Whitney *U* tests; (Placebo versus MHBA) are presented. MHBA group (n = 49); Placebo group (n = 49); *$p < 0.05$, **$p < 0.01$ (Wilcoxon signed-rank test compared with baseline). MHBA, matured hop bitter acids.

Supplementary Table 3. Mean scores of State-Trait Anxiety Inventory (STAI) and Karolinska Sleepiness Scale-Japanese version (KSS-J).

	group	baseline		week 12	
		mean ± SD	*p*	mean ± SD	*p*
**STAI-FormX**					
Anxiety-Trait	Placebo	44.0 ± 9.0	0.97	43.0 ± 9.4	0.93
	MHBA	43.9 ± 10.0		42.3 ± 8.8*	
Anxiety-State (before tests)	Placebo	42.0 ± 6.8	0.79	42.1 ± 6.9	0.72
	MHBA	42.1 ± 7.8		41.7 ± 7.0	
Anxiety-State (after tests)	Placebo	44.0 ± 9.2	0.93	40.6 ± 6.3**	0.70
	MHBA	44.0 ± 10.2		40.9 ± 8.4**	
**KSS-J**					
before tests	Placebo	2.6 ± 1.2	0.056	2.9 ± 1.5	0.24
	MHBA	3.3 ± 1.5		3.2 ± 1.5	
after tests	Placebo	3.2 ± 1.7	0.44	3.0 ± 1.7	0.49
	MHBA	3.7 ± 2.2		3.2 ± 1.6*	

Data represent mean ± SD. *p*-value from Mann–Whitney *U* tests; (Placebo versus MHBA) were presented. MHBA group (n = 49); Placebo group (n = 49); *$p < 0.05$ (Wilcoxon signed-rank test compared with baseline). MHBA, matured hop bitter acids.

Supplementary Table 4. Indicators in serum

	group	baseline		week 12	
		mean ± SD	*p*	mean ± SD	*p*
BDNF (ng/mL)	Placebo	44.73 ± 10.84	0.13	43.86 ± 10.10	0.15
	MHBA	48.38 ± 12.81		46.89 ± 10.53	
C3 (mg/dL)	Placebo	101.7 ± 15.4	0.71	100.0 ± 15.3	0.70
	MHBA	100.5 ± 16.8		98.8 ± 16.4	
TTR (mg/dL)	Placebo	26.44 ± 5.28	0.18	25.87 ± 5.24	0.048
	MHBA	27.82 ± 4.76		27.94 ± 4.99#	
ApoA1 (mg/dL)	Placebo	157.4 ± 21.0	0.42	151.7 ± 20.7**	0.68
	MHBA	154.0 ± 20.1		150.0 ± 20.9	
CCK (pg/mL)	Placebo	661.2 ± 266.7	0.17	602.0 ± 223.3*	0.15
	MHBA	732.1 ± 236.1		664.2 ± 200.8*	

Data represent mean ± SD. *p*-value from unpaired *t* test; (Placebo versus MHBA) were presented. MHBA group (n = 49); Placebo group (n = 49); *$p < 0.05$, **$p < 0.01$ (paired *t* test compared with baseline), #$p < 0.05$ (unpaired *t* test; Placebo versus MHBA). MHBA, matured hop bitter acids; BDNF, brain-derived neurotrophic factor; TTR, Transthyretin; ApoA1, Apolipoprotein AI; CCK, cholecystokinin.

Supplementary Table 5. Characteristics of the study participants at baseline (subgroup analysis)

Characteristics	SCD-P (n = 50)			SCD-C (n = 33)		
	Placebo (n = 28)	MHBA (n = 22)	*p*e	Placebo (n = 17)	MHBA (n = 16)	*p*
Age	53.7 ± 5.5	53.5 ± 5.8	0.92	52.9 ± 4.4	55.0 ± 5.0	0.22
Male/female	17/11	8/14	0.15	3/13	7/10	0.31
MMSE score (/30)	28.5 ± 1.1	28.3 ± 1.1	0.58	28.6 ± 2.0	28.3 ± 1.5	0.56
SCD-Q score (/24) ##	14.4 ± 3.9	13.4 ± 4.2	0.38	17.6 ± 4.5	20.4 ± 3.2	0.051
Employed/unemployed	23/5	18/4	1.00	13/3	13/4	1.00

Data represent mean ± SD. *p*-values were calculated unpaired *t* test, except for the male/female and employed/unemployed *p*-value, which was calculated using χ^2 test. ##*p* <0.001 (unpaired *t* test SCD-P versus SCD-Q). MHBA, matured hop bitter acids; MMSE, Mini-Mental State Evaluation; SCD-Q, subjective cognitive decline questionnaire; SCD-P, population subjective cognitive decline; SCD-C, clinical subjective cognitive decline.
